# Prevalence and determinants of HIV shedding in breast milk during continued breastfeeding among Zambian mothers not on antiretroviral treatment (ART): A cross-sectional study

**DOI:** 10.1097/MD.0000000000017383

**Published:** 2019-11-01

**Authors:** David Gatsinzi Rutagwera, Jean-Pierre Molès, Chipepo Kankasa, Mwiya Mwiya, Edouard Tuaillon, Marianne Peries, Nicolas Nagot, Philippe Van de Perre, Thorkild Tylleskär

**Affiliations:** a Centre for International Health, University of Bergen, Bergen, Norway; b Department of Pediatrics and Child Health, University Teaching Hospitals, School of Medicine University of Zambia, Lusaka, Zambia; c Pathogenesis and Control of Chronic Infections, INSERM, Université de Montpellier, Etablissement français du Sang; d University Hospital of Montpellier, Montpellier, France.

**Keywords:** breast milk, cell-associated HIV, cell-free HIV, continued breastfeeding, deoxyribonucleic acid, ribonucleic acid, sub-clinical mastitis

## Abstract

The risk of postnatal HIV transmission exists throughout the breastfeeding period. HIV shedding in breast milk beyond six months has not been studied extensively. The aim of this study was to determine prevalence and determinants of HIV shedding in breast milk during continued breastfeeding

A cross-sectional study was nested in the PROMISE-PEP trial in Lusaka, Zambia to analyze breast milk samples collected from both breasts at week 38 post-partum (mid-way during continued breastfeeding). We measured concurrent HIV deoxyribonucleic acid (DNA) and HIV ribonucleic acid (RNA) as proxies for cell-associated HIV (CAV) and cell-free HIV (CFV) shedding in breast milk respectively. Participants’ socio-demographic date, concurrent blood test results, sub clinical mastitis test results and contraceptive use data were available. Logistic regression models were used to identify determinants of HIV shedding in breast milk (detecting either CAV or CFV).

The prevalence of HIV shedding in breast milk at 9 months post-partum was 79.4% (95%CI: 74.0 – 84.0). CAV only, CFV only and both CAV and CFV were detectable in 13.7%, 17.3% and 48.4% mothers, respectively. The odds of shedding HIV in breast milk decreased significantly with current use of combined oral contraceptives (AOR: 0.37; 95%CI: 0.17 – 0.83) and increased significantly with low CD4 count (AOR: 3.47; 95%CI: 1.23 – 9.80), unsuppressed plasma viral load (AOR: 6.27; 95%CI: 2.47 – 15.96) and severe sub-clinical mastitis (AOR: 12.56; 95%CI: 2.48 – 63.58).

This study estimated that about 80% of HIV infected mothers not on ART shed HIV in breast milk during continued breastfeeding. Major factors driving this shedding were low CD4 count, unsuppressed plasma viral load and severe sub-clinical mastitis. The inverse relationship between breast milk HIV and use of combined oral contraceptives needs further clarification. Continued shedding of CAV may contribute to residual postnatal transmission of HIV in mothers on successful ART.

## 1 Introduction

National health authorities in Zambia principally promote and support breastfeeding and antiretroviral therapy (ART) as the strategy that will most likely give infants born to HIV infected mothers the greatest chance of HIV-free survival.^[1]^ The country has therefore adopted the World Health Organization (WHO) recommendations to exclusively breastfeeding (EBF) for the first six months and continued breastfeeding with supplementary foods up to one year. Thereafter, extended breastfeeding until a safe and nutritionally adequate diet can be provided for the infant.^[2]^ This is because EBF covers all nutritional and immunological requirements of the newborn baby^[3]^ and both EBF and maternal ART promotes child survival.^[4,5]^

Both CAV and CFV have been associated with postnatal transmission of HIV^[6–8]^ with CAV thought to be more important in early breastfeeding while CFV was thought to be more important in late breastfeeding.^[9,10]^ Successful maternal ART suppresses CFV but has little or no effect on CAV ^[11–13]^implying that breastfeeding infants are still exposed to CAV even in the presence of successful ART. CFV in breast milk originates partly from HIV replication in infected lymphocytes and macrophages resident in mammary glands and partly from continued seeding of systemic HIV particles across the mammary epithelial layer.^[6,14,15]^ CAV arise from local infections of mammary gland permissive cells and recruitment of HIV infected cells migrating from inducing sites of the mucosal immune system into the mammary gland and subsequently in breast milk.^[6,15]^ The mean concentration of CAV per 10^6^ BMC is lowest in colostrum^[7]^ and is thought to increase over time even in the presence of ART.^[11,12]^

Most studies looking at HIV-1 shedding in breast milk have focused on the period up to six months postpartum^[9,11,13,16–18]^ and yet a substantial proportion of late postnatal transmission of HIV-1 occurs beyond 6 months.^[19,20]^ This leaves a knowledge gap at a time developing infant is still at high risks of HIV-1 acquisition. Additionally, most studies looking at HIV shedding in breast milk consider CFV and CAV separately. Considering CFV and CAV separately has the potential to underestimate HIV shedding in breast milk since some mothers who shed CFV may not shed CAV and vice versa. The few studies that have looked at HIV shedding beyond 6 months^[7,8]^ used samples collected from one breast and report cumulative prevalence rather than point prevalence.

This study therefore seeks to determine prevalence of HIV shedding based on both breasts and both CAV and CFV in breast milk of HIV infected mothers not on ART. Our study will also explore determinants of shedding either CAV or CFV in breast milk during continued breastfeeding. This information is important as we strive to eliminate postnatal transmission of HIV since both CAV and CFV have been associated with late postnatal transmission of HIV. Additionally, we have an opportunity to study wild type HIV which would be unethical in this era of universal ART.

## 2 Materials and methods

### 2.1 Study design, setting and sample size

This was an exploratory cross-sectional study nested in the Zambian cohort of PROMISE PEP trial (NCT00640263) conducted in Burkina Faso, South Africa, Uganda and Zambia from November 16th, 2009 to May 7th, 2012. Its protocol^[21]^ and the main findings^[19]^ have been published. The main objective of the study was to evaluate the efficacy and safety of infant pre-exposure prophylaxis (PreP) using either Kaletra (Lopinavir / Ritonavir) or Lamivudine (3TC) in infants breastfeeding from HIV infected mothers. The trial included breastfeeding mothers not eligible for ART at the time and their breastfeeding infants. Mothers were asked to exclusively breastfeed for the first 6 months and thereafter continue breastfeeding with complementary foods. They were also advised to gradually wean their infants by week 49. To prevent breastfeeding transmission of HIV, infants were randomized to receive either Kaletra (Lopinavir / Ritonavir) or Lamivudine (3TC) daily up to week 50 or 1 week after cessation of breastfeeding. Potential participants were approached during antenatal visits and those willing to participate in the study were screened. After provision of informed consent, eligible mothers-infant pairs were randomized into the 2 arms of the trial (Kaletra and 3TC) at day 7 (±2) after delivery. Follow up visits were set at week 2 and monthly thereafter up to week 50. At each visit breastfeeding counseling and support was provided. Health status of both the mother and the infants were also assessed. Breast milk samples were collected at day 7, week 6, week 14, week 26 and week 38 while blood samples from the mother before delivery, day 7, week 14 and week 38. At 7, week 6, week 14, week 26 and week 38 and week 50, dry blood spots samples were collected for early infant diagnosis of HIV.

The source population for this sub study was the 563 mothers randomized into PROMISE-PEP trial in Lusaka Zambia. Only week 38 samples were included in this study as they were collected mid-way during continued breastfeeding and were the furthest available samples thereby maximizing the likelihood of detecting HIV in breast milk as participating mothers took ARV for PMTCT during pregnancy and delivery. Required sample size to determine prevalence of HIV shedding was estimated to be 229 (assuming 50% prevalence and a design effect of 1) using open source epidemiologic statistics for public health (OpenEpi) version 3.1 updated on 6th April 2013 (www.OpenEpi.com) sample size calculator.

### 2.2 Study participants and samples

Inclusion criteria for this sub study were:

a) provision of informed consent to store and use samples for future research;
b) returning for week 38 follow-up visits;
c) still breastfeeding at week 38; and
d) having week 38 breast milk samples from both breasts stored at −80°C.

All mothers who met these criteria and had no missing data (248) were included in the study

### 2.3 Sample collection and processing

Whole blood and breast milk samples were collected in the study clinic and archived in the study laboratory during the PROMISE-PEP trial. Both the clinic and the laboratory were located at the University Teaching Hospital (UTH) in Lusaka. After breastfeeding the baby and providing informed consent, mothers were asked to manually express a minimum of 10 mL breast milk, from each breast, into separate sterile 50 mL conical centrifuge tubes under supervision of a study nurse who also collected venous blood immediately after milk expression.

During PROMISE-PEP trial, whole blood processing included performing full blood, CD4 counts, Plasma viral load and storage of plasma while breast milk processing included storage of breast milk acellular fraction and breast milk cells (BMC) pellets. Storage was done at −80°C within 24 hours until further laboratory analysis. For this sub study lactoserum HIV RNA load and breast milk cell HIV DNA load were quantified as surrogate makers of cell-free HIV (CFV) and cell-associated HIV (CAV) in breast milk.

### 2.4 Quantification of HIV RNA in breast milk

To detect HIV RNA in breast milk, thawed left and right breast milk acellular fractions were centrifuged at 1200×g for 5 minutes at room temperature to separate lactoserum from lipid layer. Total RNA was extracted from 1 mL of clear lactoserum using QIAamp ultrasens virus kit (Qiagen, Hilden, Germany) and HIV-1 RNA was quantified using Generic HIV-1 charge virale real time RT-PCR kit (Biocentric, Bandol, France) and reported as copies per milliliter (copies/mL). Results were categorized as follow; ‘not detected’, ‘detected < 50 copies/mL’, ‘detected ≥ 50 copies/mL’ according to manufactures recommendations.

### 2.5 Quantification of HIV DNA in BMCs

To detect HIV DNA in BMCs thawed left and right dry breast BMC pellets were re-suspended in 200 μL of sterile DNase/RNase-free PBS. Total DNA was extracted from the re-suspended pellets using QIAamp DNA mini kit (Qiagen, Hilden, Germany) and quantified using a nanodrop spectrophotometer (Thermo Scientific, Waltham, Massachusetts). HIV-1 DNA quantified using Generic HIV DNA cell PCR kit (Biocentric, Bandol, France) and reported as copies per 10^6^ cells. Results were categorized as ‘not detected’ and ‘detected’ according to manufactures recommendations.

### 2.6 Data collection and management

Breast milk laboratory results were entered into a database and individually counterchecked to remove errors. Concurrent blood tests results, sub clinical mastitis tests results, breastfeeding and contraceptive use data were obtained from the PROMISE-PEP. A mother was considered to shed CAV if HIV DNA was detected in at least one sample from either breast. Similarly, a mother was considered to shed CFV if HIV RNA was detected in at least one sample from either breast. Mothers were considered to shed HIV if either HIV DNA or HIV RNA were detected in at least one sample from either breast. Low CD4 count was defines as having a CD4 count less than 500 cells/μL while unsuppressed plasma viral load was defined as ≥ 1000 copies/mL. SCM status was determined as negative (Na+/K+ ratio < 0.6), mild SCM (Na+/K+ ratio between 0.6 and 1) and severe SCM (Na+/K+ ratio > 1). Contraceptive data was by self-report using a questionnaire and mothers grouped as follows; oral contraceptives, injectable contraceptives and non-hormonal or no contraceptive used

### 2.7 Statistical analysis

All statistical tests were two-tailed with statistical significance at *P* < .05. Confidence level was set at 95% and power at 80%. For continuous variables either the mean with 95% confidence interval (CI) or median with inter-quartile range (IQR) were reported depending on whether the variable was normally distributed or not. Percentages were reported for categorical variables. Spearman's rho was used to measure correlation between continuous variables. Paired *t* test was used to compare means after log_10_ transformation while Pearson Chi-Square test was used to compare proportions.

Multinomial and binary logistic regression models were used to identify potential factors associated HIV shedding in breast milk. Bivariable analysis with an interaction term was done to assess interaction between variables. Multivariable logistic regression models were constructed to determine factors significantly associated with HIV shedding in breast milk. In each model, all variables associated with the dependent variables (*P* < .20) were included in final multivariable models (method: enter). Analysis was repeated using CFV shedding and CAV shedding as outcome variables to identify which component is affected. Less than 10% of eligible mothers had missing data and were excluded from the analysis. Statistical analysis was done using statistical package for social sciences (SPSS) version 20 (IBM Corporation, Armonk, New York) and open source epidemiologic statistics for public health (OpenEpi) version 3.1 updated on 06th April 2013 (www.OpenEpi.com).

### 2.8 Ethical considerations

This sub study analyzed archived samples from the PROMISE-PEP trial. The trial was approved by the University of Zambia biomedical Research Ethics Committee (IRB No. 00001131 of IORG 0000774; FWA No. 00000338). Approval for this sub study was obtained from the scientific committee of PROMISE PEP trial, ERS CONVERGE Institution Review Board (IRB No. 00005948; FWA No. 00011697) and the Regional Committee for Medical Research Ethics of Norway (REK Nord no 200802523-2) approved the study protocol. All participating mothers gave written informed consent during the main trial for storage and future use of their samples in research.

## 3 Results

### 3.1 Participants’ characteristics

During the PROMISE-PEP trial, 563 breastfeeding infants were randomized in Lusaka, Zambia,. Of the 315 mothers who were still breastfeeding at week 38, 270 had breast milk samples from both breasts stored at −80^o^C and therefore eligible to participate in this study. Of these eligible mothers, 22 were excluded from the study due to missing data resulting in a sample size of 248 mothers. Each mother contributed 2 samples (1 from each breast) resulting in 496 breast samples analyzed.

Participants’ characteristics are summarized in Table 1. Mothers participating in this study had a median age of 26.5 years (IQR: 23 – 32), a median parity of 3 (IQR: 2 – 4) and a mean CD4 count of 660 cells/μL (95%CI: 632 – 688). Although three-quarter (75.1%) had normal CD4 count (≥ 500 cells/μL), only a few (10.9%) had suppressed HIV-1 plasma viral load (< 1000 copies/mL). Among mothers with detectable plasma viral load, the mean log_10_ plasma viral load was 4.6 copies/mL (95%CI: 4.5 – 4.7).

**Table 1:**
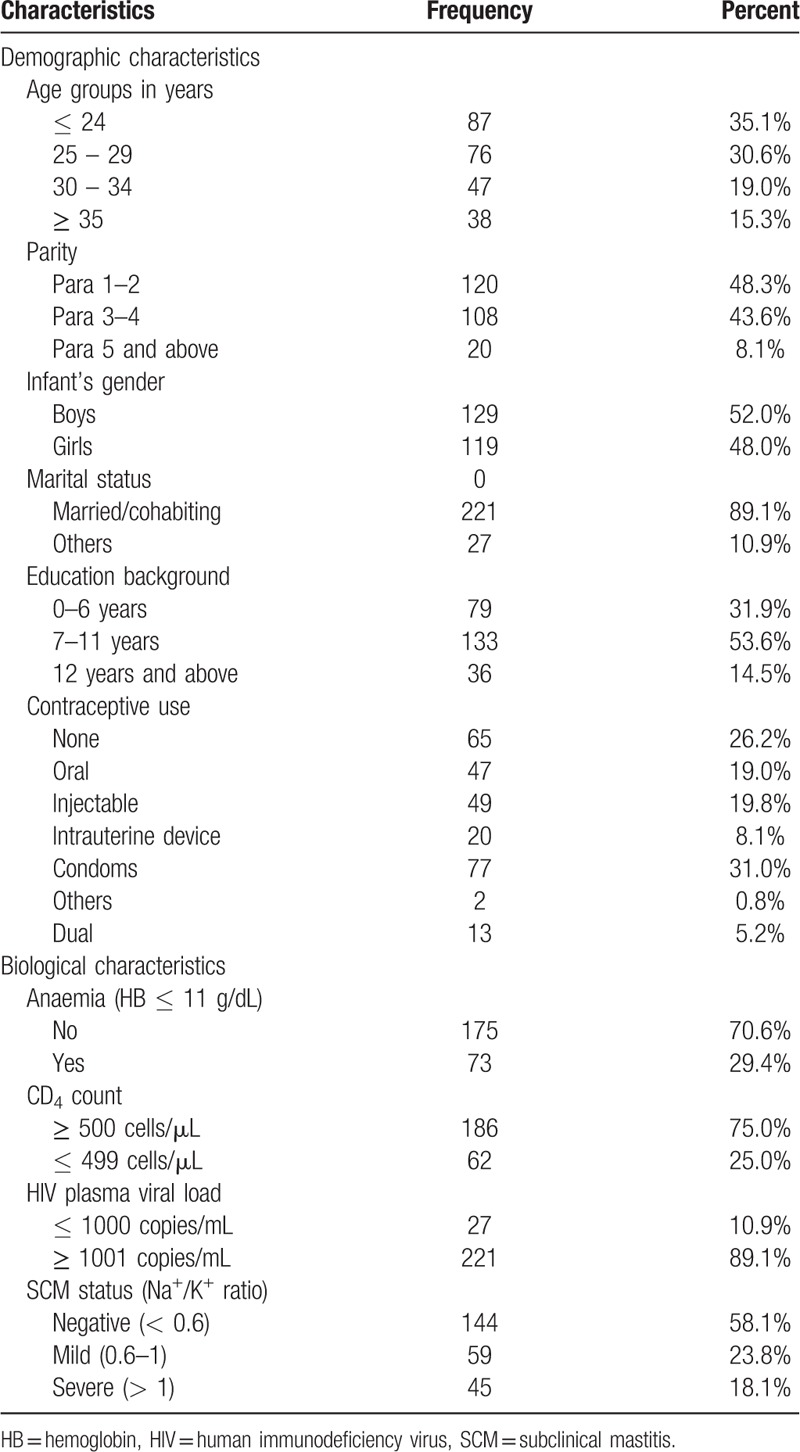
Population characteristics (n = 248).

### 3.2 HIV shedding in breast milk during continued breastfeeding

Breast milk CFV was not detected, detected < 50 copies/ml, and detected ≥ 50 copies/ml, in 85 (34.3%; 95%CI: 28.6 – 40.4), 45 (18.1%; 95%CI: 13.8 – 23.4) and 118 (47.6%; 95%CI: 41.4 – 53.8) mothers, respectively. Bilateral CFV detection (detectable in both breasts) was observed in 60.7% of the mothers with detectable CFV and there was positive correlation between CFV load in left and right breasts (r = 0.583; *P* < .001). Breast milk CFV load positively correlated with concurrent plasma viral load (r = 0.450; *P* < .001) and breast milk CAV load (r = 0.422; *P* < .001). Contrary, breast milk CFV load negatively correlated with CD4 count (r = −0.340; *P* < .001). Among samples with detectable CFV, the mean log_10_ breast milk HIV RNA load was 2.2 (95%CI: 2.1 – 2.3).

Breast milk CAV was detected in 154 (62.1%; 95%CI: 55.9 – 67.9) mothers. Bilateral CAV detection was observed in 59.7% of the mothers with detectable CAV. There was a positive correlation between CAV load in left and right breast milk samples (r = 0.348; *P* < .001). Breast milk CAV load correlated positively with plasma viral load (r = 0.198; *P* < .001) and negatively with CD4 count (r = - 0.211; *P* < .001). Among samples with detectable breast milk CAV, the mean log_10_ breast milk DNA load was 1.4 (95%CI: 1.2 – 1.5).

A total of 197 mothers had detectable CAV or CFV (shedders) translating into a 79.4% (95%CI: 74.0 – 84.0) prevalence of HIV shedding in breast milk. Of the shedders, 34 (13.7%) shed CAV only, 43 (17.3%) and the remaining 120 (48.4%) shed both CAV and CFV [120 in at least one sample (Table 2). We did not detect neither CAV nor CFV in any breast milk sample from both breasts in 20.6% (95%CI: 16.0 – 26.0) mothers (non-shedders).

**Table 2:**
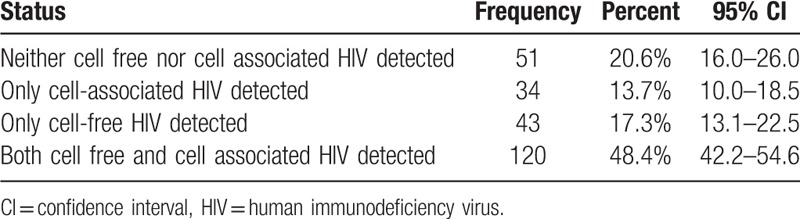
Breast milk HIV shedding categories (n = 248).

### 3.3 Determinants of HIV shedding in breast milk during continued breastfeeding

Multivariable analysis of our data show that HIV shedding (detecting either CAV or CFV) in breast milk is positively associated with low CD4 count (AOR: 3.47; 95%CI: 1.23 – 9.80), unsuppressed plasma viral load (AOR: 6.27; 95%CI: 2.47 – 15.96) and severe SCM (AOR: 12.56; 95%CI: 2.48 – 63.58). We also report an independent negative association between HIV shedding in breast milk with concurrent use of combined oral contraceptive (AOR: 0.37; 95%CI: 0.17 – 0.83) (Table 3).

**Table 3:**
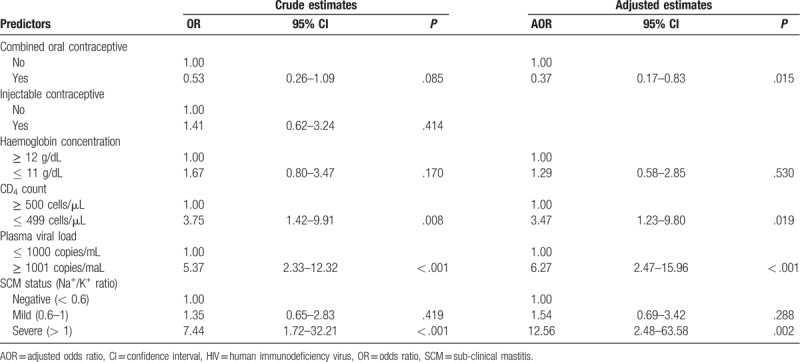
Factors associated with shedding any HIV-1 in breast milk (n = 248).

To determine factors significantly associated with each component of HIV in breast milk, we constructed separate multivariable regression models for CFV shedding (Table 4) and CAV shedding (Table 5) as outcome variables. We found that shedding CFV ≥ 50 copies/mL in breast milk was positively associated with anemia, mild SCM, severe SCM, detectable CAV, low CD4 count and unsuppressed plasma viral load. Contrary, the odds of shedding CFV ≥ 50 copies/mL was negatively associated with concurrent use of combined oral contraceptives. Last, Mothers of female infants tended to shed more CFV compared to those with male infants but the association did not reach statistical significance. On the other hand, shedding CAV in breast milk was positively associated with detecting breast milk CFV < 50 copies/mL, detecting breast milk CFV ≥ 50 copies/mL and severe SCM.

**Table 4:**
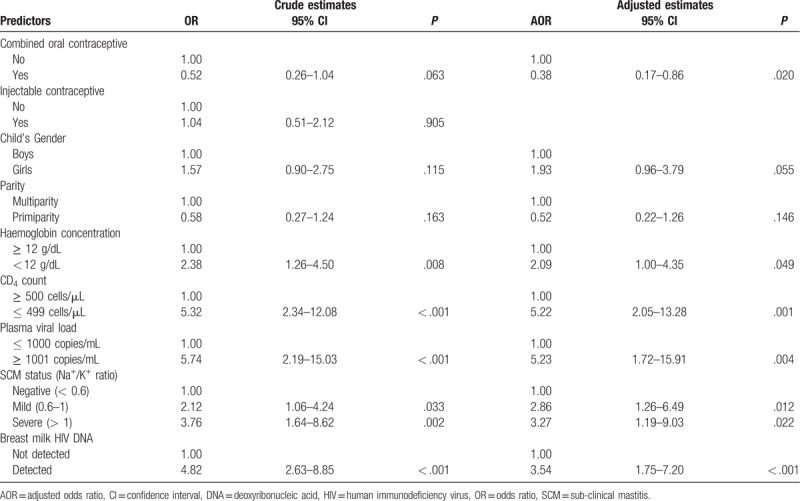
Factors associated with detection of cell-free HIV load ≥ 50 copies/mL in breast milk (n = 248).

**Table 5:**
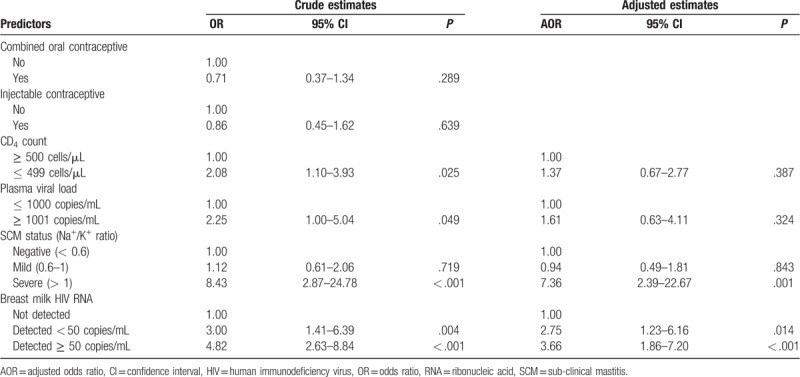
Factors associated with detection of cell-associated HIV in breast milk (n = 248).

## 4 Discussion

Our results indicate that 79.4% (95%CI: 74.0 – 84.0) of HIV infected mothers shed HIV in breast milk in the absence of ART during continued breastfeeding. As expected, the prevalence of HIV shedding in late breast milk when considering both CAV and CFV is significantly higher than prevalence estimates based on either CAV alone [62.1% (95%CI: 55.9%–67.9%) or CFV alone [65.7% (95%CI: 59.6%–71.4%), however prevalence estimates based on CAV alone and CFV alone did not differ significantly. Our estimates of CAV and CFV shedding are in line with earlier studies in the region.^[7,8]^

Our results also confirm earlier findings that CD4 count, plasma viral load, and subclinical mastitis are independently associated with HIV shedding in breast milk. In addition to these known determinants, we also found an independent significant negative association between concurrent use of combined oral contraceptive and HIV-1 shedding in breast milk. Of the identified determinants, only subclinical mastitis affects both CFV and CAV shedding and severe SCM, as an indicator of mammary gland inflammation, is by far the most important determinant HIV shedding in breast milk. Our results also indicate that whereas severe SCM is associated with both CFV and CAV, mild SCM is only associated with CFV and has no effect on CAV.

This study was done prior to the introduction of life-long ART for all in Zambia. Since 2016 all pregnant and lactating women living with HIV are started on ART for life (option B+), thus, study participants were not on ART according to national guidelines at the time. Conducting such a study currently would be unethical as all breastfeeding mothers are now supposed to be on ART. Although our results cannot be extrapolated to mothers on ART, they provide some kind of baseline data on wild type HIV that studies on mothers on ART can compare with. Our results however would apply to breastfeeding mothers still not on ART despite option B+ implementation due to suboptimal retention^[22,23]^ or poor coverage in some places.^[24]^ Latest estimates by UNAIDS reported that half of the pediatric HIV infection post-partum could be attributable to mother not on ART.

Despite not being on ART for several months (9 months), we could not detect any HIV (CAV or CAV) in breast milk samples from both breasts of about 20% mothers, suggesting some mothers naturally do not shed HIV in breast milk and therefore their infants may not be exposed to breast milk HIV. Mechanisms governing this resistance to HIV shedding in breast milk are not known. Nevertheless, our results suggest that maternal systemic HIV and sub clinical mastitis, an indicator of mammary gland inflammation, may be involved. Therefore, control of mammary gland inflammation and suppression of systemic HIV may minimize HIV shedding in breast milk.

Our results showed that in the absence of ART, there was a significant positive association between CAV and CFV suggesting local replication of HIV in the mammary gland. Indeed mammary gland CD4 cells and macrophages are more immunologically activated than their blood counterparts making them more permissive to HIV infection and replication.^[6]^ This association is disrupted in mothers on successful ART which suppresses CFV but has little or no effect on CAV^[11,12]^ explaining why studies looking at mothers on ART fail to find significant association between CAV and CFV.^[11]^

Previous studies have reported association between plasma viral load and breast milk CAV^[7,9]^ but these studies did not take into account breast milk CFV. In our study, breast milk CAV was significantly associated with plasma viral load and CD4 count in univariate analysis. Controlling of milk CFV attenuated this association thereby, indicating that the effect of systemic HIV on breast milk CAV is through breast milk CFV. Since CAV persist in breast milk beyond six months, breastfeeding infants therefore remain at risk of CAV related HIV acquisition^[6,7,9,25]^ even in the presence of successful maternal ART. Additionally, studied on breastfeeding mothers on successful ART have reported episodic detection of CFV in their breast milk over time^[11]^ which puts breastfeeding infants at risk of HIV acquisition too. Therefore, supplementing maternal ART with infant ARV prophylaxis, which is effective against both CFV and CAV^[6,19]^ mediated HIV acquisition, for the entire breastfeeding period, may be more effective in eliminating residual postnatal transmission of HIV.

Our results suggest that COC may inhibit CFV shedding in breast milk. COC contains synthetic estrogen and progestin. In our study progestin-only ICs were neither associated with CAV nor CFV in breast milk. Our results therefore, suggest that the inhibitory effect of COC on breast milk CFV may be due to estrogen. Since the rate of detecting HIV-1 in plasma (*P* = .638) did not differ significantly between COC users and non-users, our results also suggest that estrogen selectively inhibits HIV-1 in the mammary gland. Earlier studies reported a similar tissue specific inhibitory effect of estrogen on HIV-1 replication in the cervico-vaginal tract despite similar plasma viral load.^[26]^ Literature indicates that estrogen inhibits HIV-1 replication by suppressing HIV-1 long terminal repeats (LTR) promoter activity^[27,28]^ thereby inhibiting HIV-1 transcription and by reducing susceptibility of CD4+ lymphocytes and macrophages to HIV-1 infection.^[29,30]^ Further studies are needed to confirm this association.

Infant sex has not been previously associated with CFV shedding in breast milk. Although marginally significant (*P* = .055), our study suggests that mothers of female infants may shed more breast milk CFV compared to those of male infants. Recent findings indicate that infant's gender may influence the composition of human breast milk^[31–33]^ and breastfeeding practice,^[34]^ both of which would affect HIV shedding in breast milk. In line with our findings, earlier studies^[35,36]^ reported higher risks of early postnatal acquisition of HIV in female infants. This was attributed to possible higher susceptibility to HIV infection in female infants than in males arising from genetic, immunological, hormonal and environmental factors.^[35]^ Our results provide an additional mechanism to explain higher rates of postnatal HIV transmission in female infants. However, contrary to our results, a large meta-analysis^[37]^ reported significantly higher risks of late postnatal acquisition of HIV in male infants. This study, however, did not consider maternal plasma viral load thereby ignoring an important confounder to this association.

Our study had several strengths. Firstly, our study had largest number of breast milk samples collected at the same time point compared to similar studies. Secondly, HIV shedding was estimated based on both breasts and both CAV and CFV thereby giving a true reflection of HIV shedding in breast milk. Thirdly, a rigorous sampling and randomization process in the parent trial means that we had representative sample and confounders were dealt with. Lastly, mothers included in this study were not on ART therefore it would not be possible to conduct such a study currently. Limitations of our study were, firstly, this was a secondary analysis of a trial designed for a different purpose. Secondly, contraceptive use data was collected by self-report. However, this data was collected during family planning counseling at each monthly visit and therefore mothers were very familiar contraceptive methods. Thirdly, study participants were selected from a cohort of HIV-1 infected breastfeeding mothers with high CD4+ count and not on ART. Therefore, our results may not be extrapolated to mothers on ART or with low CD4 count. This being a cross sectional study, the causal nature of the association between HIV shedding and its determinants could not be assessed. Lastly wide confidence intervals for some of our estimates may indicate the need for a larger sample size to improve accuracy of our estimates.

This study estimated that about 80% of HIV infected mothers not on ART shed HIV in breast milk during continued breastfeeding. Major factors driving this shedding were low CD4 count, unsuppressed plasma viral load and severe sub-clinical mastitis. The inverse relationship between breast milk HIV and use of combined oral contraceptives needs further clarification. Continued shedding of CAV may contribute to residual postnatal transmission of HIV in mothers on successful ART.

## Acknowledgments

First and for most we would like to thank the mothers who participated in the ANRS12174 trial. We would like to thank Pr M. Laroque for providing us with access to a flame spectrometer, University Teaching Hospital; Lusaka and University of Montpellier for providing lab space and staff at the Centre for International health and UMR 1058 for their support and cooperation. We would also like to acknowledge the contribution of the ANRS 12174 trial group: ANRS 12174 trial group: University of Montpellier 1 (France): Roselyne Vallo, Valerie Marechal, Dorine Neveu, Vincent Foulongne, Michel Segondy; University of Paris V (France): Stephane Blanche, Jean-Marc Treluyer, Deborah Hirt; Makerere University (Uganda): James K. Tumwine, Grace Ndeezi, Charles Karamagi, Philippa Musoke, Proscovia M. Mugaba, Mary Kwagala, Joan Murungi, Hawa Nabuuma Muweesi, Evelyn Ninsiima, Simon Baryeija, Frederic Juma, Caleb Bwengye Kata, Stuart Katushabe; University of Ouagadougou (Burkina Faso): Nicolas Meda, Rasmata Ouedraogo, Diarra Ye, Eric Some, Hugues A. Traore, Christelle Nadembega, Justin Konate, Arsene Zongo, Abass Ouedraogo, Desire Neboua, Aissatou Belemvire, Armel Bambara, Justine Boncoungou; Danielle University of Western Cape (South Africa): Cheryl Nikodem, Justus Hofmeyr, Kim Harper, Debra Jackson, David Sanders, Mandisa Singata, Amwe Aku, Collins Okegbe-Eze, Xoliswa Williams, Nolundi Mshweshwe, Vatiswa Henge, Fikiswa Gomba, Tapiwa Gundu, Oswell Khandwa; University of Zambia (Zambia): Mildred Lusaka, Mary Chizyuka, Mary Phiri, Billies Imakando, Mwenechanya Musaku, Monica Kapasa, Gondwe Clement, Hilton Mwila Mwaba, Japhet Matoba, Chafye Siuluta, Katai Chola, Patricia Mwamutanda; University of Bergen (Norway): Halvor Sommerfelt, Ingunn Engebretsen, Jorn Klungsoyr, Jan van den Broeck, Jorn Blume; INSERM-ANRS (France): Claire Rekacewicz.

## Author contributions

**Conceptualization:** David Gatsinzi Rutagwera, Jean-Pierre Molès, Chipepo Kankasa, Mwiya Mwiya, Edouard Tuaillon, Nicolas Nagot, Philippe Van de Perre, Thorkild Tylleskär.

**Data curation:** David Gatsinzi Rutagwera, Jean-Pierre Molès, Chipepo Kankasa, Mwiya Mwiya.

**Formal analysis:** David Gatsinzi Rutagwera, Marianne Peries, Nicolas Nagot.

**Funding acquisition:** Chipepo Kankasa, Philippe Van de Perre, Thorkild Tylleskär.

**Investigation:** David Gatsinzi Rutagwera, Jean-Pierre Molès, Chipepo Kankasa, Mwiya Mwiya, Philippe Van de Perre, Thorkild Tylleskär.

**Methodology:** David Gatsinzi Rutagwera, Jean-Pierre Molès, Chipepo Kankasa, Mwiya Mwiya, Edouard Tuaillon, Marianne Peries, Nicolas Nagot, Philippe Van de Perre, Thorkild Tylleskär.

**Project administration:** David Gatsinzi Rutagwera.

**Resources:** Chipepo Kankasa, Mwiya Mwiya, Edouard Tuaillon, Marianne Peries, Nicolas Nagot, Philippe Van de Perre.

**Software:** David Gatsinzi Rutagwera, Marianne Peries.

**Supervision:** Jean-Pierre Molès, Chipepo Kankasa, Edouard Tuaillon, Philippe Van de Perre, Thorkild Tylleskär.

**Visualization:** David Gatsinzi Rutagwera.

**Writing – original draft:** David Gatsinzi Rutagwera.

**Writing – review & editing:** Jean-Pierre Molès, Chipepo Kankasa, Mwiya Mwiya, Edouard Tuaillon, Marianne Peries, Nicolas Nagot, Philippe Van de Perre, Thorkild Tylleskär.

David Gatsinzi Rutagwera orcid: 0000-0001-7092-9307.

David Gatsinzi Rutagwera orcid: 0000-0001-7092-9307.
